# *Nigella sativa* Oil Reduces LPS-Induced Microglial Inflammation: An Evaluation on *M*_1_/*M*_2_ Balance

**DOI:** 10.1155/2022/5639226

**Published:** 2022-06-14

**Authors:** Azar Hosseini, Vafa Baradaran Rahimi, Hassan Rakhshandeh, Vahid Reza Askari

**Affiliations:** ^1^Pharmacological Research Center of Medicinal Plants, Mashhad University of Medical Sciences, Mashhad, Iran; ^2^Department of Cardiovascular Diseases, Faculty of Medicine, Mashhad University of Medical Sciences, Mashhad, Iran; ^3^Applied Biomedical Research Center, Mashhad University of Medical Sciences, Mashhad, Iran; ^4^Department of Pharmaceutical Sciences in Persian Medicine, School of Persian and Complementary Medicine, Mashhad University of Medical Sciences, Mashhad, Iran; ^5^Department of Persian Medicine, School of Persian and Complementary Medicine, Mashhad University of Medical Sciences, Mashhad, Iran

## Abstract

**Objectives:**

The immune system plays a critical defence role against infections, injuries, and carcinogenic stimuli. As the macrophages of the brain resides in the innate immune system, microglia and their polarisation (*M*_1_/*M*_2_) play regulatory roles in inflammation in CNS, such as Parkinson's, Alzheimer's, dementia complex, and multiple sclerosis. *Nigella sativa* belongs to the Ranunculaceae family and has different anti-inflammatory and antioxidant effects. We conducted this study to evaluate the anti-inflammatory and protective properties of *N. sativa* oil (NSO) on the microglial cells and their polarisation (*M*_1_/*M*_2_) in the presence of LPS as a model of neuroinflammation.

**Methods:**

The protective effects of NSO (10–40 *µ*g/ml) were studied on the LPS-induced microglial cells, and the levels of tumour necrosis factor-*α* (TNF-*α*), interleukin-1*β* (IL-1*β*), IL-6, prostaglandin E_2_ (PGE_2_), and IL-10 were evaluated using both ELISA and gene expression methods. The levels of cyclooxygenase-2 (COX-2), inducible NOS (iNOS), and arginase-1 (Arg1) were also evaluated using the real-time PCR method. In addition, nitrite oxide (NO) and urea were measured using biochemical methods.

**Results:**

NSO decreased LPS-induced toxicity at all doses (*P* < 0.001). NSO (10–40 *μ*g/ml) also significantly reduced the levels of TNF-*α*, PGE2, IL-1*β,* and IL-6 in the presence of LPS (*P* < 0.01 to 0.001). Pretreatment with NSO attenuated the levels of iNOS but increased Arg1 (*P* < 0.001). The ratio of iNOS/Arg1 was also decreased in the presence of NSO (*P* < 0.001) than that of the LPS group (*P* < 0.001).

**Conclusion:**

NSO attenuated LPS-induced inflammation and increased microglia's anti-inflammatory status. These results may prove that NSO is potentially an immunomodulator for various neurodegenerative diseases by M1 phenotype dominancy, such as Alzheimer's and Parkinson's diseases.

## 1. Introduction

Generally, the immune system plays a key role in defending our body against infections and injuries as well as carcinogenic stimuli. In contrast, overactivation of the immune system leads to exceeded inflammation, regarding oxidative and inflammatory mediators, and host injuries consisting of autoimmune diseases and allergic conditions as well as cancers [1–3]. Inflammatory diseases have been growing all over the world during the past two decades, which assigned a new research line to them [4]. One of the essential inflammation research targets has ideally been arranged to the immune system and the innate immune system for attributed focal diseases, such as central nervous system (CNS) inflammatory diseases, including Parkinson's and Alzheimer's diseases [4–6].

Microglia are an essential part of the brain's innate immune system and play crucial regulatory roles in CNS inflammation [7–9]. Upon stimulation with a plethora, microglial cells get activated and begin a cascade of inflammation in the CNS. These cells also contribute to the pathogenesis of some diseases, such as Parkinson's disease, Alzheimer's disease, AIDS, dementia complex, multiple sclerosis, and ischemia [10–12]. Following the microglia activation, the liberation of some inflammatory mediators, including nitric oxide (NO), inducible NO synthase (iNOS), interleukin (IL)-1*β*, IL-6, and tumour necrosis factor-*α* (TNF-*α*) is increased in the CNS microenvironments, in which they possess an important role in proceeding neurodegenerative diseases [9, 12, 13].

Lipopolysaccharides (LPS), by acting on toll-like receptor-4 (TLR-4), lead to the release of proinflammatory and neurotoxic agents and differentiate macrophages into inflammatory type 1 macrophages (*M*_1_) [14–19]. Given that this type of macrophages deserves an essential role against injuries and noxious stimuli, including bacterial and viral infections as well as tumour cells, overactivation of the cells causes inflammatory diseases, such as neurodegeneration, rheumatoid arthritis, multiple sclerosis, and other autoimmune diseases [5, 16, 17]. In contrast, type 2 macrophage (*M*_2_) cells, which are also known for their action as healing macrophages, produce anti-inflammatory mediators consisting of IL-10 and IL-4 as well as highly expressed arginase 1 (Arg-1) to provide urea from the catabolism of arginine [6, 12, 16, 17].

*Nigella sativa* belongs to the Ranunculaceae family and grows in Southwest Asia. *N. sativa* has been considered as an herbal medicine in Islamic culture and in Avicenna's famous book, Qanun [20]. In this regard, it has been mentioned that the seeds of *N. sativa* act as traditional remedies for the treatment of different neurological-based diseases, such as memory impairment, epilepsy, pain, and neurotoxicities, as well as Alzheimer's (AD) and Parkinson's (PD) diseases [20, 21]. Phytochemical analysis has reported the presence of different chemical compounds in *N. sativa,* such as phospholipids [22], fatty acids [23], vitamins [24], and ascorbic acid [25]. Also, other compounds have been found in *N. sativa,* including dithymoquinone, thymoquinone (TQ), thymol, and carvacrol, which have therapeutic effects as an analgesic [26], antioxidant [27], and anticancer as well as immune-modulatory activities [28–30]. Furthermore, recent studies have reported anti-inflammatory effects of *N. sativa* extract and its active compounds on different inflammation models, such as rheumatoid arthritis in rat models [31], eicosanoid generation in leukocytes [32], allergic lung inflammation in a mouse model [33], carrageenan-induced paw oedema [34], ulcerative colitis [35], mouse dendritic cells [36], allergic encephalomyelitis as an animal model for multiple human sclerosis [37], and nitric oxide production by murine macrophages [38].

LPS-induced microglia activation is an appropriate model for *in vitro* investigation to understand related mechanisms that play a considerable role in CNS inflammatory disease [6, 39]. Furthermore, due to the importance of microglial activation during neuroinflammatory diseases, regulating these cells can be considered a therapeutic pathway. Moreover, in the light of the knowledge that *N. sativa* concomitantly suggested in traditional medicines and experimental and pharmacological experiments, we conducted the current *in vitro* study to evaluate the direct anti-inflammatory and protective properties of *N. sativa* oil (NSO) on the microglial cells' polarisation (*M*_1_/*M*_2_) in the presence of LPS as a model of neuroinflammation.

## 2. Materials and Methods

### 2.1. Chemicals, Reagents, and Kits

DMEM/F12 media culture, penicillin plus streptomycin (pen/strep), amphotericin B, FBS, DMSO, Ficoll, DNAse I, Dispase II, LPS, and other cell culture materials were purchased from Sigma-Aldrich Chemical Co. (St. Louis, MO, USA). RBC lysis buffer (10x, Cat No: 420301) was purchased from Biolegend company (San Diego, CA, USA). Proliferation assay kit (MTT) and ELISA kits (PGE2, IL-6, IL-1*β*, IL-10, and TNF-*α*) were purchased from Roche Diagnostics (Mannheim, Germany) and eBioscience (San Diego, CA, USA), respectively. All the other materials were of analytical and cell culture grade and were prepared from Sigma-Aldrich Chemical Co. (St. Louis, MO, USA).

### 2.2. Preparation of *N. sativa* Seed Oil Extract (NSO)

*N. sativa* seeds were purchased from the local market in Mashhad, Iran. 10 g of the dried and powdered seeds were weighed, and its oil was prepared using a cold press with no solvent or heat exposure. The pressing process was carried out at a temperature lower than 35°C in chrome-nickel cold press oil squeezing machines (Household Oil Press, Oily® YD-ZY-03A, Germany). The oil was filtered through 100% cotton filters with a thickness of 2 mm, which was free from dense particles and kept at −20°C until use. The efficiency was 29.5% w/w, which is due to the weight of the dried leaves. 50 mg of extract was dissolved in 1 ml of complete DMEM/F12 (10% FBS + 1% Pen/Strep) plus 1% DMSO to prepare stock at 50 mg/ml concentration. Experimental concentrations were prepared from this stock with DMEM/F12 enriched and 10% FBS and 1% Pen/Strep. The final level of DMSO was lower than 0.1% v/v for tested concentrations.

### 2.3. Mice Microglia Isolation and Cell Culture

Primary microglial cells were prepared in accordance with the previously described Lee and Tansey method [12, 14, 17, 18]. Microglia cells were cultured in DMEM/F12 plus 1% v/v of Pen/Strep (100×) and 10% v/v of heat-inactivated FBS supplemented with 0.5 *µ*g/mL amphotericin B and 2 mL glutamine. Cells were maintained in a humidified incubator at 37°C and 5% v/v CO_2_.

### 2.4. Protocol of the Experimental Procedure

Inflammatory condition on microglia was induced by the addition of LPS (1 µg/ml). The following groups indicate study groups:

- Group one: microglia + vehicle (control group)
- Group two: microglia + LPS (1 µg/ml)
- Group three: microglia + highest concentration of NSO (40 *µ*g/ml)
- Groups four to seven: microglia + NSO (10, 20, and 40 *µ*g/ml) + after two hours LPS (1 µg/ml)

The final concentration of DMSO was lower than 0.1% v/v for all experiments.

### 2.5. Cell Proliferation Assay

The effects of various concentrations of NSO were examined in the presence or absence of LPS on microglia. In brief, about 5000 cells were seeded in a 96-well plate and treated with NSO (0–160 *µ*g/ml) and NSO (0–160 *µ*g/ml) + LPS (1 *µ*g/ml) and incubated for 48 hours at 37°C in a 5% v/v CO_2_ incubator. After 48 hours, 10 *μ*l of MTT reagent (5 mg/ml) was added to each well, which was incubated for the next three hours. Formazan crystals were dissolved in 100 *μ*L DMSO, and the absorbance was read using a StatFAX 2100 ELISA plate reader (Awareness Inc, USA) at 570 nm in reference with 620 nm.

### 2.6. Cytokine Assays

Indexing of the inflammatory state induced by LPS and inflammatory cytokines including TNF-*α*, IL-1*β*, and IL-6, as well as PGE2, were examined using the sandwich ELISA method based on the manufacturer's instructions. The microglial cells were cultured at a density of 10^6^ cells per 6-well plate and incubated with different concentrations of NSO, based on the experimental design section. The supernatant was collected after 48 hours of incubation, and various cytokines were measured.

### 2.7. Quantitative Real-Time PCR (qRT-PCR)

To evaluate the effect of different concentrations of NSO on gene expression, the levels of related mRNA of TNF-*α*, IL-1*β*, IL-6, COX-2, iNOS, and Arg1 were also examined. GAPDH was considered the housekeeping gene. The primer sequences are mentioned in Table 1. The relative quantity of each mRNA was normalised to the relative quantity of GAPDH mRNA. The PCR conditions were as follows: 95°C for three minutes, followed by 40 cycles of 95°C for 30 seconds, 55°C for 30 seconds, and 72°C for 30 seconds, respectively [14, 40]. The values for gene expression levels were examined using the ΔCt method, and fold-change values were reported as 2^−(ΔΔCt)^.

### 2.8. Nitric Oxide and Urea Assay

The amount of nitrite was measured as an indicator of the concentration of the produced nitric oxide by using the method of Griess as described previously [41]. The supernatants, which were collected for cytokines assay, were used to examine the concentration of nitrite produced by the microglia cells at 540 nm using Griess reagent (G4410 SIGMA) in a spectrophotometer. The nitrite concentration was determined using the sodium nitrite standard curve [1]. Based on the manufacturer's instructions, urea was also detected using a colorimetric assay kit from Abcam (catalogue no. ab83362).

### 2.9. Statistical Analysis

Data were prepared as means ± SEM and analysed by GraphPad Prism® 6 software (GraphPad Software, San Diego, CA). Comparisons between groups were performed by using a one-way analysis of variance (ANOVA) with the Tukey–Kramer *post hoc* test. The significance was approached at *P* < 0.05, <0.01, and 0.01, respectively. Raw data were also reported in Tables 2 and 3.

## 3. Results

### 3.1. Effects of NSO on Cell Viability with or without LPS

**Figure 1:**
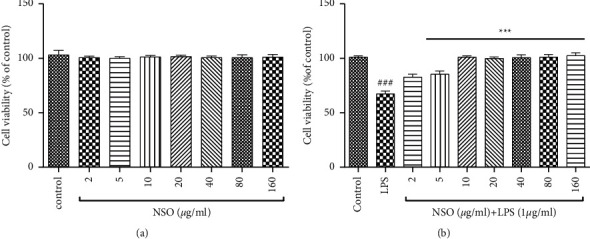
Effects of NSO on cell viability without (a) or with (b) LPS. The cell viability was evaluated in the presence of NSO alone (a), and cells were pretreated with different concentrations of NSO for 2 hours and then exposed to LPS (1 *µ*g/mL) for 48 hours (b). The cell viability was quantitated by MTT assay. Results are mean ± SEM (*n* = 6).^###^*P* <  0.001 versus control; ^*∗∗∗*^*P*<  0.001 versus LPS.

The cells were incubated with different concentrations (2–160 *µ*g/ml) of NSO for 48 hours, and cell viability was evaluated. The MTT assay showed NSO alone did not decrease cell viability (Figure 1(a)). As shown in Figure 1(b), cell viability decreased in the presence of LPS (*P* < 0.0001). NSO decreased LPS-induced toxicity in all doses (*P* < 0.001).

### 3.2. Effects of NSO on TNF-*α*, PGE_2_, IL-1*β*, and IL-6 in the Presence of LPS

**Figure 2:**
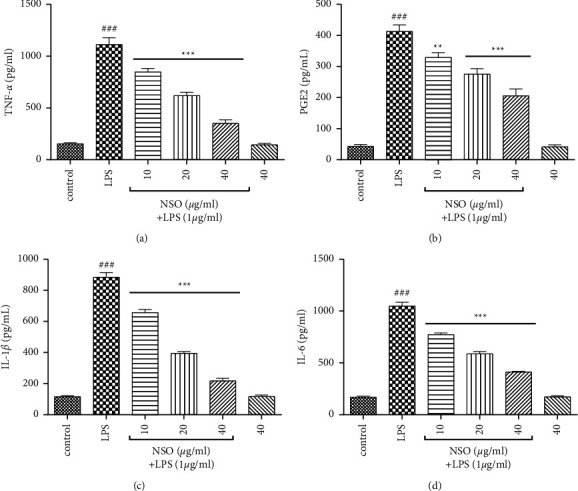
Effects of NSO on the levels of TNF-*α*, PGE_2_, IL-1*β*, and IL-6 in the presence of LPS; the cells were pretreated with NSO for 2 hours and then incubated with LPS for 48 hours. After 48 hours, the levels (pg/ml) of TNF-*α*, PGE2, IL-1*β*, and IL-6 were determined in the presence of LPS. ^###^*P* <  0.001 versus control; ^*∗∗*^*P* <  0.01 and ^*∗∗∗*^*P* <  0.001 versus LPS.

The cells were pretreated with NSO (10–40 *µ*g/ml) for two hours, and then LPS was added. After 48 hours, TNF-*α*, PGE_2_, IL-1*β*, and IL-6 were measured by using ELISA. In comparison with control, LPS significantly increased TNF-*α* (*P* < 0.001), PGE2 (*P* < 0.001), IL-1*β* (*P* < 0.001), and IL-6 (*P* < 0.001) in the supernatant. As shown in Figures 2(a)–2(d), NSO (10–40 *μ*g/ml) significantly reduced TNF-*α* (*P* < 0.001), PGE2 (*P* < 0.01 to 0.001), IL-1*β* (*P* < 0.001), and IL-6 (*P* < 0.001) in all of the doses in the presence of LPS.

### 3.3. Effects of NSO on Gene Expression of TNF-*α*, COX-2, IL-1*β*, IL-6, iNOS, and Arg1 and Ratio of iNOS/Arg1 in the Presence of LPS

**Figure 3:**
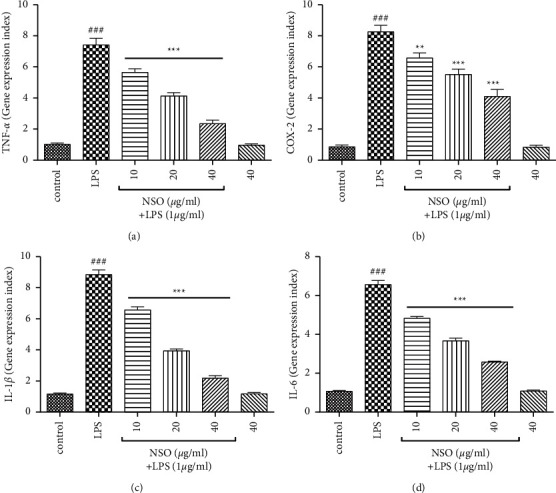
Effects of NSO on the gene expression levels of TNF-*α*, COX-2, IL-1*β*, and IL-6 in the presence of LPS; the cells were pretreated with NSO for 2 hours and then incubated with LPS for 48 hours. After 48 hours, the expression levels of TNF-*α*, COX-2, IL-1 *β*, and IL-6 were determined in the presence of LPS. ^###^*P* <  0.001 versus control; ^*∗∗*^*P* <  0.01 and ^*∗∗∗*^*P* <  0.001 versus LPS.

As shown in Figures 3(a)–3(d), LPS increased the gene expression of TNF-*α* (*P* < 0.001), COX-2 (*P* < 0.001, *P* < 0.01), IL-1*β* (*P* < 0.001), and IL-6 (*P* < 0.001). NSO (10–40 *μ*g/ml) significantly reduced the expression of TNF-*α* (*P* < 0.001), COX-2 (*P* < 0.001, *P* < 0.01), IL-1*β* (*P* < 0.001), and IL-6 (*P* < 0.001) than that of the LPS group.

**Figure 4:**
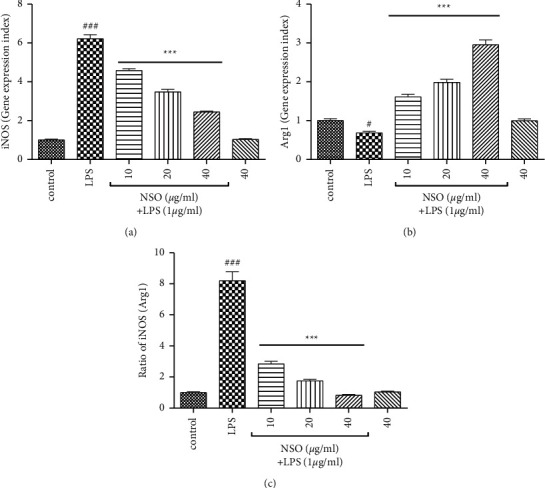
Effects of NSO on the expression of iNOS, Arg1, and the ratio of iNOS/Arg1 in the presence of LPS; the cells were pretreated with NSO for 2 hours and then were incubated with LPS for 48 hours. After 48 hours, the expression levels of iNOS, Arg1, and the ratio of iNOS/Arg1 were measured in the presence of LPS. ^#^*P* <  0.05, ^###^*P* <  0.001 versus control; ^*∗∗∗*^*P* <  0.001 versus LPS.

As illustrated in Figures 4(a)–4(c), LPS elevated the expression of iNOS (*P* < 0.001), decreased Arg1 (*P* < 0.05), and increased the ratio of iNOS/Arg1 (*P* < 0.001) than that of the control group. Pretreatment with NSO (10–40 *μ*g/ml) attenuated the levels of iNOS (*P* < 0.001) ,while it increased Arg1 (*P* < 0.001). The ratio of iNOS/Arg1 was also decreased in the presence of NSO (*P* < 0.001) than that of the LPS group (Figures 4(a)–4(c)).

### 3.4. Effects of NSO on NO, Urea, and Ratio of NO/Urea

**Figure 5:**
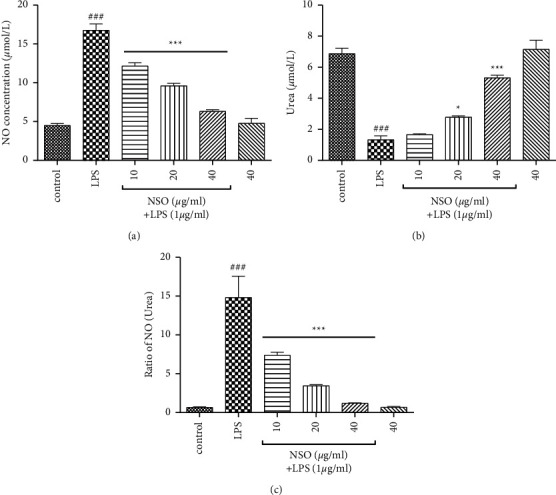
Effects of NSO on NO, urea, and ratio of NO/urea in the presence of LPS; the cells were pretreated with NSO for 2 hours and then were incubated with LPS for 48 hours. After 48 hours, the levels of NO, urea, and ratio of NO/urea were measured in the presence of LPS. ^###^*P* <  0.001 versus control; ^*∗*^*P* <  0.05 and ^*∗∗∗*^*P* <  0.001 versus LPS.

As shown in Figures 5(a)–5(c), LPS increased NO (*P* < 0.001), decreased urea (*P* < 0.001), and elevated the ratio of NO/urea (*P* < 0.001). In contrast, NSO (10–40 *μ*g/ml) reduced the level of NO (*P* < 0.001), increased urea (*P* < 0.05 and *P* < 0.001), and decreased the ratio of NO/urea (*P* < 0.001) than that of the LPS group.

## 4. Discussion

Activation of microglial cells has an essential role in the pathogenesis of some neuroinflammation diseases, such as Alzheimer's disease, Parkinson's disease, and MS [15, 16]. The induction of these cells leads to the secretion of proinflammatory cytokines, such as TNF-*α*, various ILs, prostaglandins, leukotrienes, and NO [42, 43]. These factors lead to neuroinflammation and cell death in neurons. Therefore, the antioxidant and anti-inflammatory agents may protect neuronal cells via inhibition of microglial activation [44, 45].

Alzheimer's disease (AD), the most incident age-related neurodegenerative disorder with cognitive impairment, is characterised by progressive brain atrophy and the presence of intracellular neurofibrillary tangles consisting of hyperphosphorylated tau protein and extracellular senile plaques composed of amyloid-beta (A*β*) peptide and neuroinflammation [46]. Moreover, A*β* plaques form in the extracellular space, thus illustrating the most obvious opportunity for direct targeting and activating by microglia [47]. In this study, we investigated the protective and anti-inflammatory effects of NSO on LPS-induced inflammatory responses in microglial cells. The studies have shown that activation of microglia leads to the production of neurotoxic factors, such as NO, which iNOS synthesises. NO at low concentrations has protective effects, but at higher levels, it is a neurotoxic agent via nitrite-free radical production [48].

Microglia might also aid in reducing A*β*, a process that has been extensively studied and reviewed [47]. Microglia (*M*_2_), by secreting proteinases, such as neprilysin, insulin-degrading enzyme, and MMP9, directly degrade and clean soluble A*β*. Generally, microglia are presumed to phagocytose and degrade A*β*. The scavenger receptor CD36 and TREM2 has been proposed as direct phagocytic receptors for A*β* or A*β*-lipoprotein complexes that direct the lysosomal degradation of A*β* [47]. Several studies indicated that *N. sativa* and its main bioactive constituent, thymoquinone, provide neuroprotection and attenuated disease results in AD animal models. Neuroprotective role of NSO and its fractions: hexane, ethyl acetate, and water from *N. sativa* were also investigated in primary cells against A*β*-induced cytotoxicity, which is a common characteristic of AD. Oil and water-fractionated, treated primary rat cerebellar granule neuron (CGN) cells treated with 3‐(4,5‐dimethylthiazol‐2‐yl)‐5‐(3‐carboxymethoxyphenyl)‐2‐(4‐sulfophenyl)‐2H‐tetrazolium, and inner salt (MTS) demonstrates higher cell viability. Hexane and ethyl acetate fractions show higher antioxidant properties against 10 *µ*M A*β*_1-40_-treated primary cells [46]. Our findings showed that LPS reduced cell viability, increased TNF-*α*, IL-1*β*, IL-6, PGE_2_, iNOS, NO, and the ratio of iNOS/Arg1 and NO/urea. At the same time, NSO reduced all indexes in microglia cells and decreased LPS-induced inflammation. Recent studies have shown that callus (0.2 to 1.6 mg/ml) and seed (1.25 to 20 *μ*l/ml) extracts of *N. sativa* reduced inflammation in rat glial cells by lowering nitric oxide [49]. Also, *N. sativa* and *N. sativa* oil decreased inflammation in rats that received LPS [50].

Parkinson's disease (PD) was firstly reported in 1817 by James Parkinson, and the second most prevalent neurodegenerative disorder with cognitive and motor deficits is characterised by a gradual loss of dopaminergic neurons in the substantia nigra pars compacta. Reactive astrocytes are present in AD and PD and around active demyelinating lesions in multiple sclerosis. Experimentally, to understand the *N. sativa* fatty acid function on the suborganellar level, mitochondrial membrane potential was studied in MMP^+^-induced apoptotic PC12 cells, which shows the protective role of fatty acids [51]. Also, *N. sativa* seeds prevented LPS toxicity in peritoneal macrophages by modifying NO and iNOS expressions [52]. In addition, a clinical study showed the consumption of 500 mg of *N. sativa* oil as a capsule by rheumatoid arthritis patients decreased MDA and NO [53]. Thymoquinone reduced A*β*-induced neurotoxicity in SK-N-SH cells via inhibition of NF-*κ*B-p65 and reversed the expression of MAP2 [54]. Another study revealed that thymoquinone reduced LPS-toxicity in activated BV-2 microglial cells by decreasing cytokines [55]. Different mechanisms can play a role in the anti-inflammatory activity of *N. sativa* and thymoquinone, such as inhibition of NF-*κ*B activation and its molecular targets [36, 56], which causes the reduction of inflammation in neurons, also attenuation of cytokines including TNF-*α*, IL-1*β* [31], nitric oxide (NO)/iNOS, IL-6, IFN-*γ*, prostaglandin E2 [57], TGF-*β*1 [58], 5-lipoxygenase activity [59], and cyclooxygenase-2 [60]. All of these studies confirmed our study and the anti-inflammatory effect of *N. sativa*. Therefore, this herb and its active compounds may be regarded as an anti-inflammatory drug in the future.

Although the cytokine profiles in this manuscript are roughly consistent with the phenotype shift of *M*_1_/*M*_2_, the polarisation of macrophages is generally recognised to be driven by transcription factors [61]. However, polarisation cannot be considered a fixed model, while macrophages sufficiently reserve the plasticity to integrate multiple signals, such as microbes, injured tissues, and the tissue environment [61]. In particular, two prototypic transcription factors, such as NF-*κ*B and STAT3, are broadly involved in proinflammatory regulation [62, 63]. Therefore, as a limitation of the current study, we did not measure the transcriptional factors for confirmation of the *M*_1_/*M*_2_ polarisation. Eventually, we suggest the measurement of them in further experimental assessments.

## 5. Conclusion

In conclusion, the study revealed that NSO had no cytotoxicity effect on microglial cells. In addition, NSO significantly attenuated inflammatory responses of LPS and increased the anti-inflammatory status of microglia by regulating the *M*_1_/*M*_2_ ratio towards the *M*_2_ state. These results may prove that NSO is potentially an immunomodulator for various neurodegenerative diseases by *M*_1_ phenotype dominancy, such as Alzheimer's and Parkinson's diseases.

## Figures and Tables

**Table 1:** The primers for real-time PCR.

Gene	Forward primer (5′-3′)	Reverse primer (5′-3′)	Ref.
Arg*1*	CATGGGCAACCTGTGTCCTT	TCCTGGTACATCTGGGAACTTTC	[19]
*iNOS*	GACGAGACGGATAGGCAGAG	GTGGGGTTGTTGCTGAACTT	[19]
TNF-*α*	CATCTTCTCAAAATTCGAGTGACAA	TGGGAGTAGACAAGGTACAACCC	[64]
IL-1*β*	GGAGAACCAAGCAACGACAAAATA	TGGGGAACTCTGCAGACTCAAAC	[40]
IL-6	GTTTTCTGCAAGTGCATCATCG	GGTTTCTGCAAGTGCATCATCG	[40]
COX-2	GCTGCCCGACACCTTCAACATT	CACATTTCTTCCCCCAGCAACC	[40]
GAPDH	GGAGGAACCTGCCAAGTATG	TGGGAGTTGCTGTCCTGGGCTGCACT	[40]

**Table 2:** The raw data (mean ± SEM) for MTT assays in the presence and absence of LPS stimulation.

	Control	LPS	NSO (*μ*g/ml)						
			2	5	10	20	40	80	160
MTT									
Mean	103.2	—	100.7	100.2	101.3	101.7	100.8	100.8	101.2
SEM	4.102	—	1.256	1.376	1.202	1.282	1.327	2.330	2.386

MTT + LPS									
Mean	101.2	67.47	82.82	85.56	101.2	100.0	100.8	101.2	102.5
SEM	1.138	2.415	2.631	2.816	1.138	1.342	2.330	2.386	2.513

**Table 3:** The raw data (mean ± SEM) for ELISA and gene expression and biochemical assays in the presence of LPS stimulation.

	Control	LPS	LPS + NSO (*μ*g/ml)			NSO (*μ*g/ml)
			10	20	40	40
PGE_2_						
Mean	43.50	413.3	329.3	276.2	205.8	42.17
SEM	5.536	20.67	15.26	17.09	21.70	5.952

TNF-*α*						
Mean	154.2	1113	847.5	621.2	353.2	145.0
SEM	11.73	64.11	33.85	30.51	32.57	13.14

IL-1*β*						
Mean	117.2	885.0	658.2	395.0	219.0	118.2
SEM	5.759	29.90	19.17	11.50	14.67	8.332

IL-6						
Mean	171.0	1050	773.8	588.2	413.3	174.8
SEM	6.826	34.13	13.88	20.77	6.427	7.414

TNF-*α* gene						
Mean	1.000	7.420	5.650	4.141	2.354	0.9667
SEM	0.07821	0.4274	0.2257	0.2034	0.2171	0.08760

COX-2 gene						
Mean	1.000	8.267	6.587	5.523	4.117	0.8433
SEM	0.1107	0.4133	0.3053	0.3419	0.4341	0.1190

IL-1*β* gene						
Mean	1.000	8.850	6.580	3.948	2.190	1.182
SEM	0.05759	0.2990	0.1920	0.1152	0.1467	0.08332

IL-6 gene						
Mean	1.000	6.560	4.837	3.675	2.583	1.093
SEM	0.04272	0.2135	0.08645	0.1298	0.04017	0.04633

iNOS gene						
Mean	1.000	6.220	4.586	3.485	2.449	1.036
SEM	0.04050	0.2024	0.08196	0.1231	0.03809	0.04393

Arg-1 gene						
Mean	1.006	0.6854	1.614	1.986	2.958	0.9970
SEM	0.04350	0.03525	0.06462	0.07948	0.1184	0.04415

iNOS/Arg-1						
Mean	1.000	8.200	2.850	1.750	0.8333	1.050
SEM	0.05164	0.5727	0.1648	0.09574	0.04216	0.04282

NO						
Mean	4.500	16.75	12.17	9.625	6.333	4.792
SEM	0.2661	0.8490	0.4014	0.2940	0.2108	0.6338

Urea						
Mean	6.877	1.336	1.660	2.788	5.319	7.154
SEM	0.3452	0.2399	0.05658	0.08570	0.1708	0.5853

NO/urea						
Mean	0.6648	14.83	7.382	3.465	1.185	0.6824
SEM	0.05456	2.726	0.3716	0.1325	0.06169	0.08993

## Data Availability

The data used to support the findings of this study are available from the corresponding author upon reasonable request.

## Abbreviations

AD: Alzheimer's disease
AGS: Adenocarcinoma gastric cells
Arg1: Arginase 1
CAT: Catalase
CD14: Cluster of differentiation 14
CNS: Central nervous system
COX-2: Cyclooxygenase-2
DSS: Dextran sulfate sodium
GSH: Glutathione
IL-1*β*: Interleukin-1*β*
IL-6: Interleukin-6
IL-10: Interleukin-10
IFN-*γ*: Interferon-gamma
iNOS: Inducible nitrite oxide synthase
LPS: Lipopolysaccharide
MS: Multiple sclerosis
MDA: Malondialdehyde
MTT: 3-(4,5-Dimethylthiazol-2-yl)-2,5-diphenyl tetrazolium bromide
NF-*κ*B: Nuclear factor *κ*B
NO: Nitrite oxide
PD: Parkinson's disease
PHA: Phytohemagglutinin
PGE2: Prostaglandin E2
SIF: Stock isotonic Ficoll
SOD: Superoxide dismutase
UC: Ulcerative colitis
TNF-*α*: Tumour necrosis factor-*α*
WHO: World Health Organization.

## Additional Points

Declaration of transparency and scientific rigour: this declaration acknowledges that this paper adheres to the principles for transparent reporting and scientific rigour of preclinical research recommended by funding agencies, publishers, and other organisations engaged with supporting research.
